# Naturalist's Monthly Report

**Published:** 1811-04

**Authors:** 


### NATURALIST'S MONTHLY REPORT.

### FEBRUARY.

### *Thawing Month.*

——————The rivers swell,
Of bonds impatient, sudden from the hills,
O'er rocks, and woods, in broad brown cataracts,
A thousand snow-fed torrents shoot at once.

During the whole of the present month the weather has been extremely changeable; and on eighteen of the twenty-eight days, there has been rain, viz. on the 1st, 2d, 6th, 7th, 8th, 9th, 10th, 11th, 12th, 15th, and from the 21st to the 28th, inclusive. On the 15th, the rain was very heavy, and continued so, almost without intermission, through the whole day.—There was a frost in the night of the 16th, but it only continued until the following morning.

On

On the 1st the wind was south-west; in the afternoon of the 2d, south-east; on the 3d and 4th south-west; 5th and 6th, south-east; 7th and 8th, south-west; 9th, easterly; from the 10th to the 14th, westerly; on the 15th, east; 16th, northerly; 17th, 18th, 19th, southerly; 20th, north-west; 21st, south-south-west; 22d, and 23d, south-west; 24th, South-east, and south; 25th, 26th, and 27th, west; and on the 28th, south-west. We had strong gales on the 1st, 3d, 8th, and 26th, but particularly on the 8th.

February 3. There was a peculiarly high tide, without any apparent cause; but in the course of the day a heavy gale blowing from the south-west accounted for it.

Garden Peas begin to appear out of the ground.

February 4. A species of *Podura* or Spring-tail is come out of its place of concealment, and runs about in the day time upon old mossy walls. These insects, like all others of the same tribe, have a kind of elastic tail, which is folded under the body, and by means of which they are enabled to leap to very considerable distances. This power is evidently given to them for the purpose of aiding their escape from enemies. It is a singular circumstance that one species of these insects, (viz. *Podura aquatica* of Linneus) is found usually on the surface of the water, on which it leaps with nearly as much agility as the others do on hard substances.

February 4. The Lark and Redbreast sing. On sunny and sheltered banks, vegetation has begun. The leaves of several plants have shot out of the ground in the course of the last seven or eight days.

February 5. There are now a great number of lambs on the commons, and in pastures.

From this day to the 14th, the weather was in every respect so unfavourable, that I was scarcely able to walk out in the fields during the whole interval. I had, however, an opportunity of remarking that the leaf-buds of the gooseberry and lilac were becoming green.

February 14. The Larch aud Yew are both in flower.

Hitherto there has only been one salmon caught in our rivers during the present month.

I observed a dark coloured butterfly this day, but it was at such a distance that I could not discover the species. It was doubtless either *Papilio Io*, or *urticæ*.

The flowers of Laurustinus die and drop off. Coltsfoot *(Tussilago, farfara)* and Ivy-leaved Speedwell *(veronica hederæfolia)* are both in flower.

February 16. Rooks are beginning to pair.

The catkins of two or three species of willows appear; and the leaves of Cuckoo pint *(arum maculatum)* spring from the ground.

February 17th. Bees of different kinds are busily employed apparently in collecting the pollen from the catkins of the willows. Small insects are flying about in every direction.

The Blackbird and Thrush sing.

The season is not a forward one. The late severe frosts, and the succession of wet weather since, have tended very considerably to check the progress of vegetation.

February

February 20th. In sheltered gardens Hyacinths and Mezereon are in flower.

February 26th. Rooks are employed in forming their nests; and Partridges begin to pair.

The buds of trees begin to swell. Daffodils are in flower.

February 27th. Water Crow-foot (*Ranunculus aquatilis*) is cut for the purpose of feeding cattle

*Hampshire.*

